# Virtual screening and drug repositioning of FDA-approved drugs from the ZINC database to identify the potential hTERT inhibitors

**DOI:** 10.3389/fphar.2022.1048691

**Published:** 2022-11-18

**Authors:** Hasan Afzaal, Reem Altaf, Umair Ilyas, Shaiq Uz Zaman, Syed Damin Abbas Hamdani, Saifullah Khan, Hajra Zafar, Mustafeez Mujtaba Babar, Yongtao Duan

**Affiliations:** ^1^ Henan Provincial Key Laboratory of Children’s Genetics and Metabolic Diseases, Children’s Hospital Affiliated to Zhengzhou University, Zhengzhou University, Zhengzhou, China; ^2^ Department of Pharmaceutics, Faculty of Pharmaceutical Sciences, Riphah International University, Islamabad, Pakistan; ^3^ Department of Pharmacy, Iqra University, Islamabad, Pakistan; ^4^ Department of Basic Medical Sciences, Shifa College of Pharmaceutical Sciences, Shifa Tameer-e-Millat University, Islamabad, Pakistan; ^5^ Institute of Biotechnology and Microbiology, Bacha Khan University Charsadda, Charsadda, Pakistan; ^6^ School of Pharmacy, Shanghai Jiao Tong University, Shanghai, China

**Keywords:** hTERT inhibitors, virtual screening, drug repurposing, molecular dynamic simulation, anticancer

## Abstract

The length of the telomeres is maintained with the help of the enzyme telomerase constituting of two components, namely, a core reverse transcriptase protein (hTERT) and RNA (hTR). It serves as a significant and universal cancer target. *In silico* approaches play a crucial role in accelerating drug development processes, especially cancer drug repurposing is an attractive approach. The current study is aimed at the repurposing of FDA-approved drugs for their potential role as hTERT inhibitors. Accordingly, a library of 2,915 sets of FDA-approved drugs was generated from the ZINC database in order to screen for novel hTERT inhibitors; later on, these were subjected to molecular docking analysis. The top two hits, ZINC03784182 and ZINC01530694, were shortlisted for molecular dynamic simulation studies at 100 ns based on their binding scores. The RMSD, RMSF, Rg, SASA, and interaction energies were calculated for a 100-ns simulation period. The hit compounds were also analyzed for antitumor activity, and the results revealed promising cytotoxic activities of these compounds. The study has revealed the potential application of these drugs as antitumor agents that can be useful in treating cancer **a**nd can serve as lead compounds for further *in vivo, in vitro,* and clinical studies.

## Introduction

To date, cancer remains to be a major cause of death globally despite the availability of multiple approaches for its prevention and therapy. The major side effects limiting the use of several approaches, including radiotherapy and chemotherapy, are the deaths of not only the malignant cells but also the normal cells. About 85% of cancer cells express the human telomerase reverse transcriptase (hTERT) that is involved in the maintenance of immortality in cancer cells, which is, however, absent in most normal somatic cells (Harley et al., 1990; Meyerson et al., 1997). Telomeres are the tandem repeats of the TTAGGG sequence and are present at the distal ends of human chromosomes, functioning to stabilize the chromosomal ends and preventing chromosomal degradation. With each continual cell division, the telomeres undergo progressive shortening in somatic cells, while in immortal cells, a specialized DNA polymerase called telomerase resynthesizes and maintains the telomere length (Harley et al., 1990; Blackburn, 1991; Greider, 1991; Keith et al., 2002; Liu et al., 2004). hTERT is the major determinant of the telomerase activity and is the catalytic subunit of human telomerase. Evidence suggests the immortalization of cancer cells is achieved through the activation of telomerase expression (Nakamura et al., 1997; Weinrich et al., 1997; Bodnar et al., 1998).

The current malignant tumor therapies include mainly surgery, radiotherapy, or chemotherapy, as well as combinations of these approaches. However, these approaches have their limitations in the eradication of tumor cells. New strategies for the development of hTERT-based therapies have acquired importance as an ideal therapeutic target in human cancers with a broad therapeutic window (Lü et al., 2012). Targeting the hTERT could be a potential target in the treatment of cancer progression.

The *in silico* methods have facilitated greatly in the development of drugs for several diseases, including drug repositioning, reducing the cost, and time of discovery. The computational techniques, such as the system biology approach, virtual screening, and molecular dynamic simulation studies, have helped in identifying several potential drug targets for proteins specific for combating a disease (Ilyas et al., 2020; Altaf et al., 2021; Altaf et al., 2022a; Altaf et al., 2022b; Huang et al., 2022). Several computational approaches are available for drug repurposing. However, the results are not as accurate as those obtained in *in vivo* studies, but the reduced cost and accuracy justify the efforts. Analyzing 1,000 drugs through *in silico* repurposing consumes a much lesser amount of time than the fraction of time it takes for *in vivo* studies for the same number of drugs (Kashyap and Datta, 2022; Mathpal et al., 2022). With this advantage of drug repurposing, this study identifies potential drug targets for hTERT by obtaining a library of 2,915 compounds from the ZINC database. The main aim of this study was to screen hTERT inhibitors. The library of drugs was subjected to virtual screening against the target protein. The candidate drugs were shortlisted based on their docking scores. The top two drugs with the lowest binding energies were selected for further *in vitro* evaluation of anticancer activity against the cancer cell line HepG2. The molecular dynamics simulation study was also carried out to analyze the stability of proteins and the protein–ligand complex.

## Methodology

### Preparation of the ligand library

A library consisting of 2,915 clinical drug compounds was retrieved from the ZINC database website in mol2 format (Irwin and Shoichet, 2005) and were subjected to molecular docking analysis against the hTERT protein by PyRx software (Dallakyan and Olson, 2015). The mol2 format files were converted to pdb format using Open Babel (version 2.3.1).

### Protein structure

The x-ray crystallographic structure of the hTERT protein was obtained from the protein data bank with PDB ID: 6d6v. The protein was prepared before being subjected to molecular docking analysis by removing all the ligands, metal atoms, and water molecules from the crystal structure using Discovery Studio 4.0. The telomerase catalytic core was selected as a binding site in the protein structure for the study. The quality of protein was assessed using the QMEAN (Qualitative Model Energy ANalysis) tool of the Swiss model. QMEAN describes the global quality of the models and was found to be –8.4. The resolution of the protein was 4.1 Å.

### Structure-based virtual screening

In order to find the potential drug targets for the hTERT protein, a molecular docking analysis was carried out by screening the conformations of ligands having high affinity at the binding site of the hTERT active site by PyRx software (GUI version 0.8). Docking was performed by keeping the target protein rigid while the ligands were kept flexible. The ligands were shortlisted based on the protein–ligand complex with the lowest binding energy and were later subjected to molecular dynamic simulation studies.

### Visualization

The 2D protein–ligand interactions were analyzed using Discovery Studio 4.0 (Studio, 2008), while the 3D visualization was performed by PyMOL, a molecular visualization tool, to analyze the hydrogen bonds and the bond length (DeLano and Bromberg, 2004).

### Docking validation

The docking procedure was validated to ensure the binding efficiencies of the ligand and protein by redocking the obtained pose on the same active binding site of the protein. All the docking protocols were kept unchanged, and the grid parameters were constant. The validation of docking is based on the rmsd of the ligand at the active binding site having less deviation when compared to the actual complex. Discovery Studio 4.0 and PyMOL 2.3 were used to superimpose the redocked complex on the reference complex. The root mean square deviation was calculated.

### Molecular dynamics simulation

The structural stability of the protein and the protein–ligand complexes in physiological conditions is determined using molecular dynamic simulation studies. For this purpose, the top compounds with the lowest binding energies were used for MD simulations. In molecular dynamic simulation studies, the atom’s movements are calculated over a period of time employing Newton’s classical equation of motion. This is in contrast to molecular docking, which only provides a static view of the ligand in the active binding of proteins and predicts the ligand–protein binding status. The simulation studies were performed using the Desmond (2012) module of Schrodinger software. The top two compounds with the lowest binding energy scores were selected for molecular dynamic simulation studies at 100 nanoseconds using the OPLS-2005 force field. To predict the ligand–protein binding status, the physiological environment was used. The Protein Preparation Wizard of Maestro was employed to preprocess the ligand–protein binding complex and to optimize and minimize the complexes. The ligand–protein complex was bound in a predefined TIP3P water model in an orthorhombic box, and the overall charges of the system were neutralized by adding Na^+^ and Cl^-^ ions. The pressure was kept constant at 1.0132 bar, and the temperature was set at 300K, keeping the box volume minimized. The stability of the simulation studies was evaluated by the root mean square deviation (RMSD) for all the trajectories. (Release, 2014; Altaf et al., 2022b) (Release, 2014; Altaf et al., 2022b) (Release, 2014; Altaf et al., 2022b) (Release, 2014; Altaf et al., 2022b) (Release, 2014; Altaf et al., 2022b) (Release, 2014; Altaf et al., 2022b) (Release, 2014; Altaf et al., 2022b) (DeLano and Bromberg, 2004; Altaf et al., 2022b) (DeLano and Bromberg, 2004; Altaf et al., 2022b) (DeLano and Bromberg, 2004; Altaf et al., 2022b) (DeLano and Bromberg, 2004; Altaf et al., 2022b) (DeLano and Bromberg, 2004; Altaf et al., 2022b) (DeLano and Bromberg, 2004; Altaf et al., 2022b) ^5c, 11 5c, 11^

### Brine shrimp assay

Samples were prepared by dissolving 50 mg of adapalene (labeled A) in 5 ml of methanol (solution A1A). Solution A1B was prepared by diluting 0.5 ml of A1A to 10 ml with methanol. Ten shrimps were transferred to each sample vial using a disposable dropper, and artificial sea water was added to make it up to 5 ml. A drop of freshly prepared dry yeast suspension (3 mg in 5 ml of artificial sea water) was added as food to each vial. Appropriate amounts of solution (100 μl B, 50 μl A, and 500 μl A for 10, 100, and 1,000 mg/ml, respectively) were added to individual test vials. The control vial had no added test drug. The vials were maintained at an optimum temperature. Survivors were counted after 24 h. The same procedure was followed for tamsulosin (labeled T).

### *In vitro* cytotoxic assay

#### Cell line

The cell line (Hep G2; catalogue# HB-8065) employed in this study was obtained commercially from ATCC, United States. The HepG2 cells were cultured in low-glucose Dulbecco’s modified Eagle’s medium (LG-DMEM; Sigma-Aldrich, USA) supplemented with 10% fetal bovine serum (FBS; Sigma-Aldrich, USA), 100 IU/ml penicillin, and 100 μg/ml streptomycin (Sigma Aldrich, USA) at 37°C and 5% CO_2._

#### XTT Assay

To evaluate the cytotoxic effect of samples against cancerous cells, 2, 3-bis (2-methoxy-4-nitro5- sulphoxyphenyl)-2H-tetrazolium 5-carboxyanilide inner salt (XTT) assay was performed, according to the manufacturer’s instructions (Roche, Switzerland). In brief, 4 × 10^3^ cells/well were seeded into 96-well plates and allowed to grow for the next 24 h. After that, cells were subjected to a two-fold diluted fraction of the given samples (as instructed), and plates were kept at 37°C in an atmosphere of 5% CO_2_ for 24 h. On completion of treatment, media was aspirated, and cells were washed with phosphate-buffered saline (PBS) and incubated with 100 μl/well of a mixture of XTT and electron-coupling reagent (50:1). The absorbance was recorded after 24, 48, and 72 h at 450 nm with a reference wavelength of 650 nm by using a spectrophotometer (SpectraMax PLUS 384, USA). The details were followed as provided and mentioned in Table 1, where A1 is the active form of adapalene, A2 is the formulation of adapalene, T1 is the active form of tamsulosin, and T2 is the formulation of tamsulosin.

**TABLE 1:** Standard and tested compounds used in the study.

Plate (Time points: 24, 48, and 72 h)	
1	Negative control (NC)
2	Positive control (PC)-methotrexate
3	DMSO control (DC)
4	A-1
5	A-2
6	T-1
7	T-2

#### Calculations: two-fold dilution

For all samples, a two-fold dilution was prepared in a low-glucose serum-free medium (LG-SFM) by diluting it 50% to the original. For example, for 200 µl of the received sample, we added 200 µl of LG-SFM to make it two-fold-diluted. Similarly, for 1 ml of the sample, a two-fold dilution was prepared by adding 1 ml of LG-SFM.

## Results

### Molecular docking analysis

About 2,915 compounds were docked against the target protein hTERT. The compounds were ranked based on their docking scores. The binding energies of the top 10 are given in Table 2, which shows all the compounds having the lowest binding energies. In this study, we focused on the top two compounds showing the highest binding affinity of –11.1 kcal/mol.

**TABLE 2:** Docking scores of top 10 hit FDA-approved drugs.

Compound ID	Docking score	Molecular formula	Compound name
ZINC03784182	–11.1	C28H28O3	Adapalene
ZINC01530694	–11.1	C20H28N2O5S	Tamsulosin
ZINC03932831	–11	C27H30F6N2O2	Dutasteride
ZINC14880001	–10.9	C27H30F6N2O2	Dutasteride
ZINC01550499	–10.8	C22H22F3N	Cinacalcet
ZINC03978005	–10.8	C34H41N5O8S	Dihydroergotamine mesylate
ZINC19360739	–10.6	C26H28Cl2F2N2	Flunarizine dihydrochloride
ZINC01530886	–10.5	C33H30N4O2	Telmisartan
ZINC14880002	–10.5	C34H41N5O8S	Dihydroergotoxine
ZINC03784182	–11.1	C28H28O3	Adapalene

### Molecular interaction analysis of the top two compounds

**FIGURE 1:**
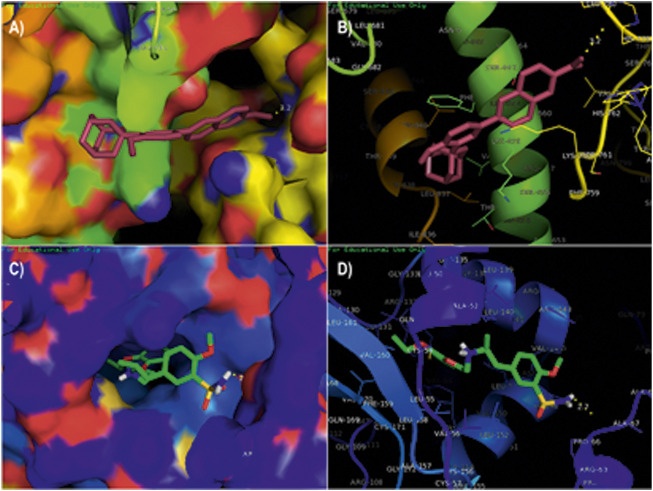
Molecular docking analysis of the top two ligands against the target protein hTERT. **(A)** Surface interaction of the ligand adapalene in the active binding site of the hTERT protein. **(B)** Molecular interaction of adapalene showing amino acid residues involved in the interaction. **(C)** Surface interaction of ligand tamsulosin in the active binding site of hTERT protein. **(D)** Molecular interaction of tamsulosin showing amino acid residues involved in the interaction.

**FIGURE 2:**
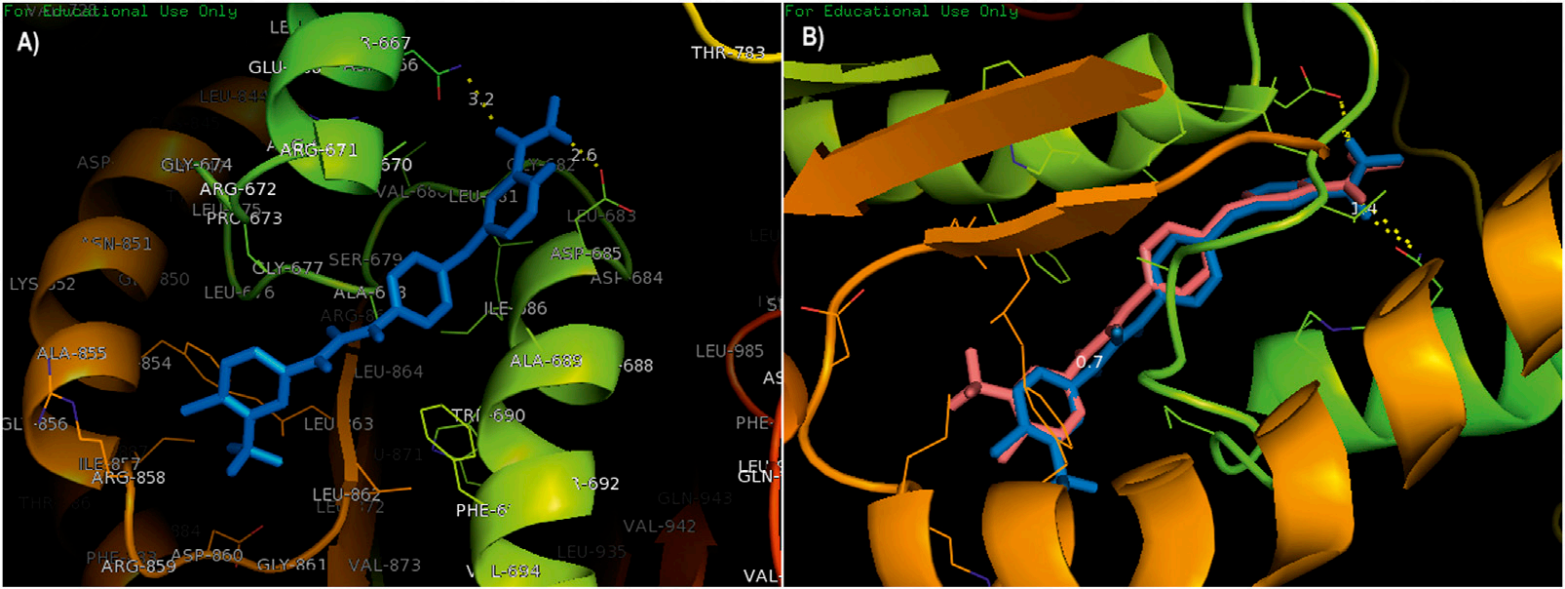
**(A)** Standard drug sorafenib was used to compare the binding energies and interaction pattern with those of the tested drugs. **(B)** Validation of docking; **t**he superimposition of a native co-crystallized ligand in the docked complex with the help of PyMOL.

The compounds adapalene (ZINC03784182) and tamsulosin (ZINC01530694) were selected for further protein–ligand interaction analysis to analyze the binding sites and the amino acid residues involved in the formation of bonds with the ligands. Discovery Studio 4.0 and the PyMOL software were used to visualize the protein–ligand interaction. The compound adapalene showed one conventional hydrogen bond with LEU780; some pi-anion and attractive charges were also observed with HIS762 and ARG742. Pi-sigma interaction was observed with PHE662, and alkyl and pi-alkyl interactions were observed with LYS659 and LEU840. The pi-hydrogen donor bond was observed with SER663. The binding analysis of tamsulosin was also observed and showed two stable conventional hydrogen bonds with PRO66 and CYS54, a carbon–hydrogen bond with ARG143, and p-pi T-shaped and amide pi-stacked interactions were observed with TRP137 and ALA136. Some alkyl and pi-alkyl interactions were also observed with LEU140, LEU153, LEU152, VAL144, VAL148, VAL51, and VAL160 (Figure 1). The standard drug sorafenib was used to compare the binding energies and interaction patterns of the tested drugs. The standard sorafenib showed the lowest binding energy of –9.6 kcal/mol. The amino acid interaction analysis revealed that three conventional hydrogen bonds were formed with ASN666, ASP685, and ALA678. Some alkyl and pi-alkyl bonding was observed with PHE854, ALA689, PRO673, and LEU863 (Figure 2A).

### Validation of docking

The validation of docking protocols and the assurance of docking efficiencies were performed by redocking the already docked pose in the active binding site of the target protein. For this purpose, the standard drug sorafenib was used to validate the procedure. The redocked pose showed binding at the same active site as that of the reference pose, with an rmsd of 0.7 kcal/mol. The interacting amino acids were LEU681, ASP685, and ASN666 showing conventional hydrogen bonding, while the amino acids ALA689, ALA678, and PHE854 showed pi-alkyl and pi-sigma interactions. The superimposition was performed using a native co-crystallized ligand in the docked complex with the help of PyMOL. A low rmsd of 0.7 kcal/mol suggested the validation of the docking procedure (Figure 2B). The blue color shows the reference conformation that was obtained during the docking procedure. The pink complex represents the redocked complex of the active compound in the active binding site of α-glucosidase.

### Molecular dynamic simulation

#### RMSD analysis

**FIGURE 3:**
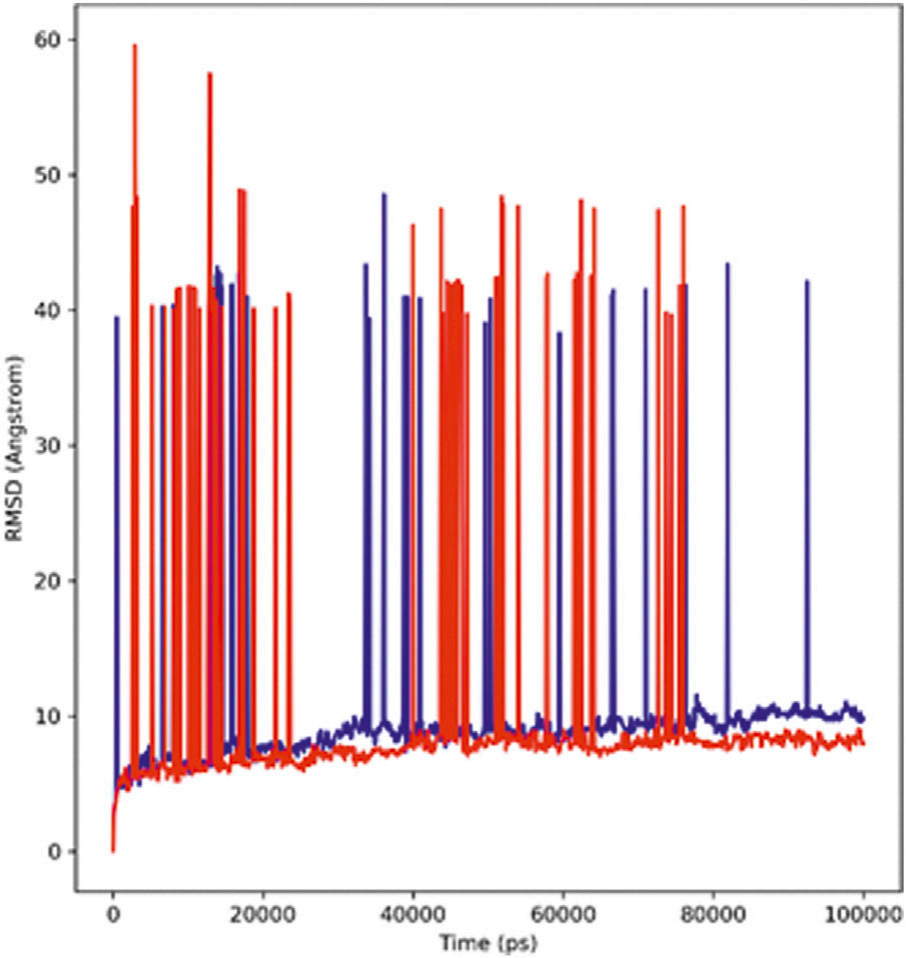
RMSD profiles of the hTERT–adapalene complex (red) and the hTERT–tamsulosin complex (blue) for 100 ns of MD simulation.

With the help of the root mean square deviation (RMSD), the changes in the protein–ligand complex were determined at 100-ns molecular dynamic simulation. The RMSD helps in determining the equilibration, flexibility of protein, and the average distance between the protein atoms. Figure 3 depicts the root mean square deviation plot showing the complexes’ stable form. The complex system showed stability throughout the simulations calculated for a 100-ns trajectory. The average RMSD calculated for the hTERT–adapalene complex was found to be 9.6 Å (red). A slight fluctuation was observed between 10 and 30 ns which then achieved equilibrium after 45 ns and remained stable. The average RMSD calculated for hTERT–tamsulosin was 9.7 Å (blue). A similar fluctuation pattern was observed over 10–30 ns, which was then equilibrated after 45 ns and remained stable throughput simulation. A low RMSD value and the few fluctuations were indicative of system stability (Kuzmanic and Zagrovic, 2010). The results suggested stable interactions and acceptable range for the studied complexes during the MD trajectories.

#### RMSF analysis

**FIGURE 4:**
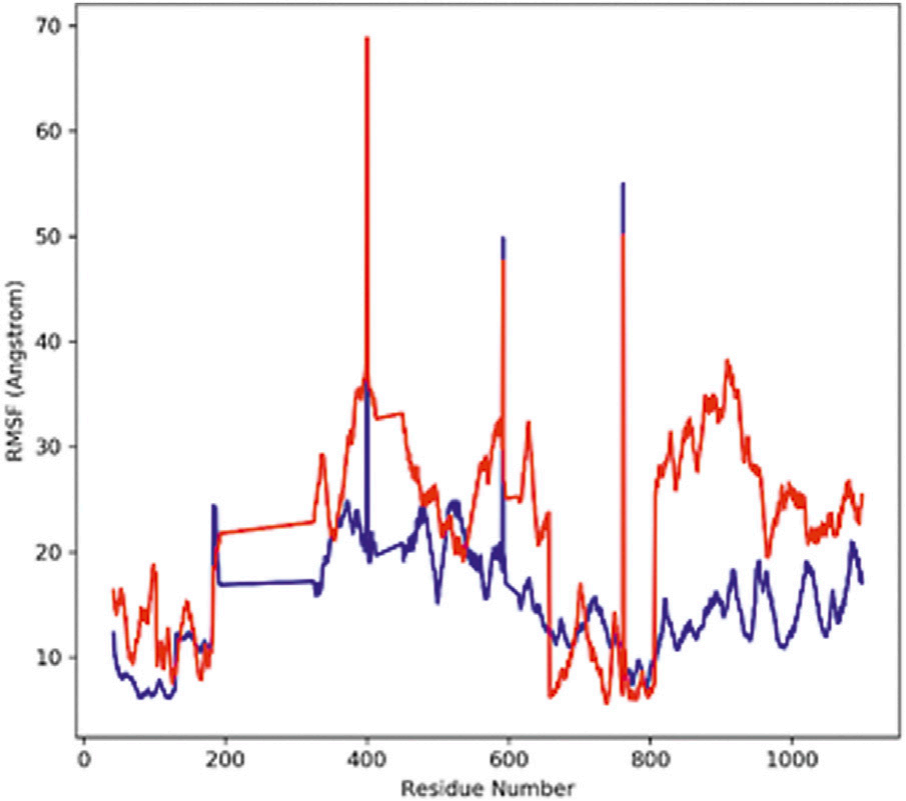
Graph displays the RMSF values of Cα atoms for 100-ns trajectories.

Root mean square fluctuation analysis (RMSF) is performed to analyze the flexible region of the protein or to study the regions of structures that fluctuate in relation to the overall structure of the protein. It measures the average movement of atoms at a specified temperature and pressure. The good stability of the system is predicted by a low RMSF value; a high value indicates greater flexibility of the system during the MD simulation. The root mean square fluctuation plot was plotted to observe the fluctuation in the residues for protein–hTERT and complexes during the 100-ns trajectory period. The flexibility of residues in the proteins and the complexes was also analyzed (Figure 4). The RMSF of both complexes showed less fluctuation and good stability.

#### Radius of gyration

**FIGURE 5:**
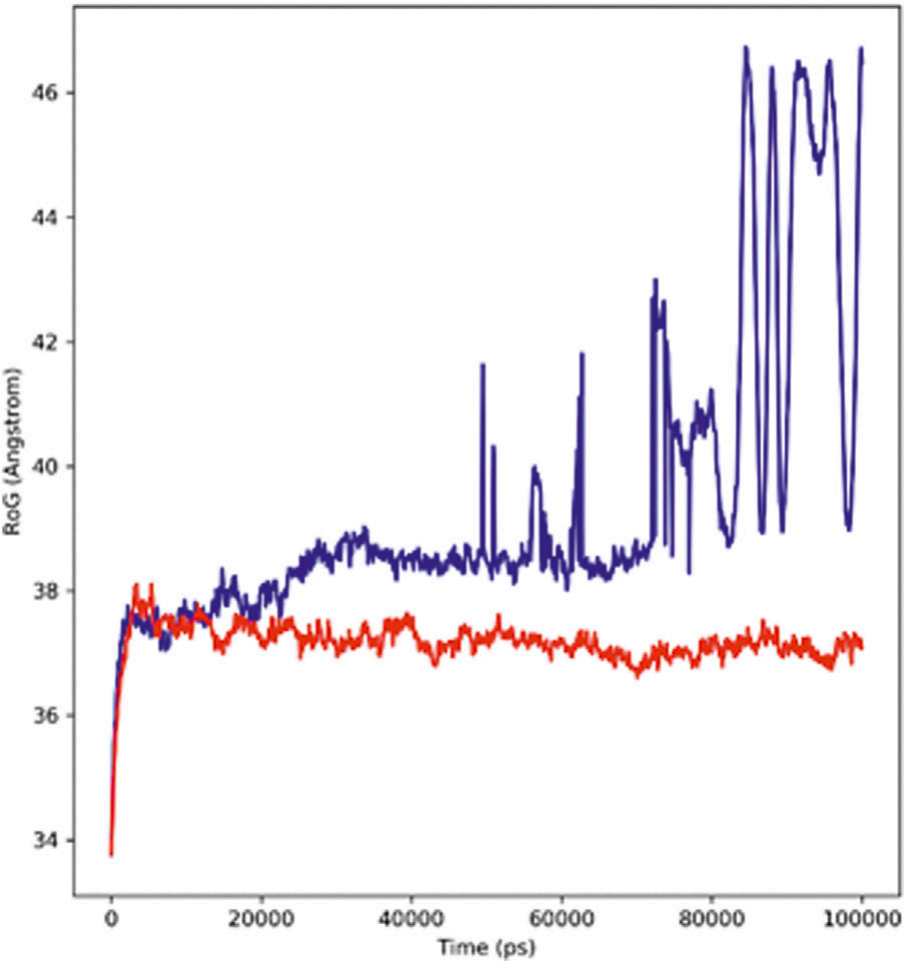
Radius of the Gyration plot showing changes observed in the conformational behavior of protein–ligand complexes (red depicting the hTERT–adapalene complex, while blue indicating the hTERT–tamsulosin complex) for 100-ns MD trajectories.

The radius of gyration (Rg) defines the stable folding and unfolding of complexes during the MD simulation. It assesses the changes in compactness between the ligand and the protein complex; if there is a higher Rg value, the compactness of the protein–ligand complex would be lower. The adapalene Rg showed an average Rg of 39.39 Å ranging from 33.7 to 46.48 Å (red), while tamsulosin Rg showed an average Rg of 37.17 Å ranging from 33.76 to 37.25 Å (blue) (Figure 5). If a relatively steady value of Rg is maintained by the protein during the MD simulation, it indicates the protein has stably folded; however, a change in Rg over time indicates the protein has unfolded (Ghasemi et al., 2016). The results indicate consistent values for Rg, and the complex showed similar behavior in terms of compactness, implying good stability.

#### Solvent accessible surface area

**FIGURE 6:**
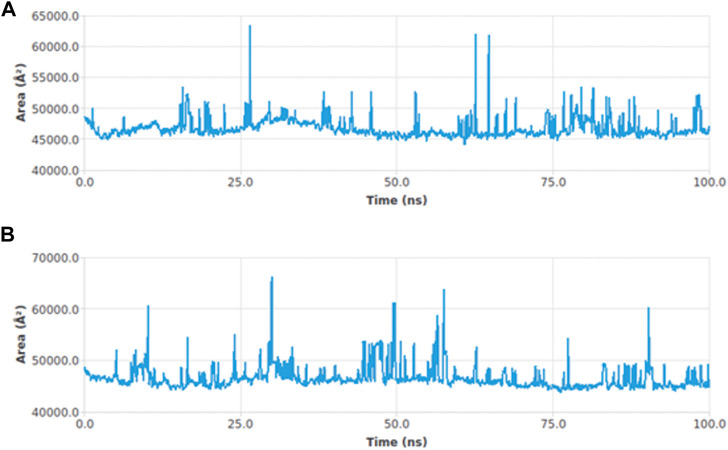
SASA curves highlighting the changes in the solvent accessibility of the studied protein complexes hTERT–adapalene **(A)** and hTERT–tamsulosin **(B)**) during 100-ns MD trajectories.

The interactions between the complexes and the solvents are measured through the solvent accessible surface area (SASA). In order to predict the conformational changes occurring during the interaction, the SASA of the protein–ligand complex was calculated. Figure 6 displays the SASA plot values verses time for both protein–ligand complexes, i.e., adapalene and tamsulosin. The average SASA value for the hTERT–adapalene complex was found to be 46800.76 nm^2^ (red); similarly, the average SASA value for the hTERT-tamsulosin complex was 46612.98 nm^2^ (blue). These calculations indicate that the SASA values for these two protein–ligand complexes are relatively stable over a period of 100 ns with no significant changes in the protein structure.

#### Interaction energy

**FIGURE 7:**
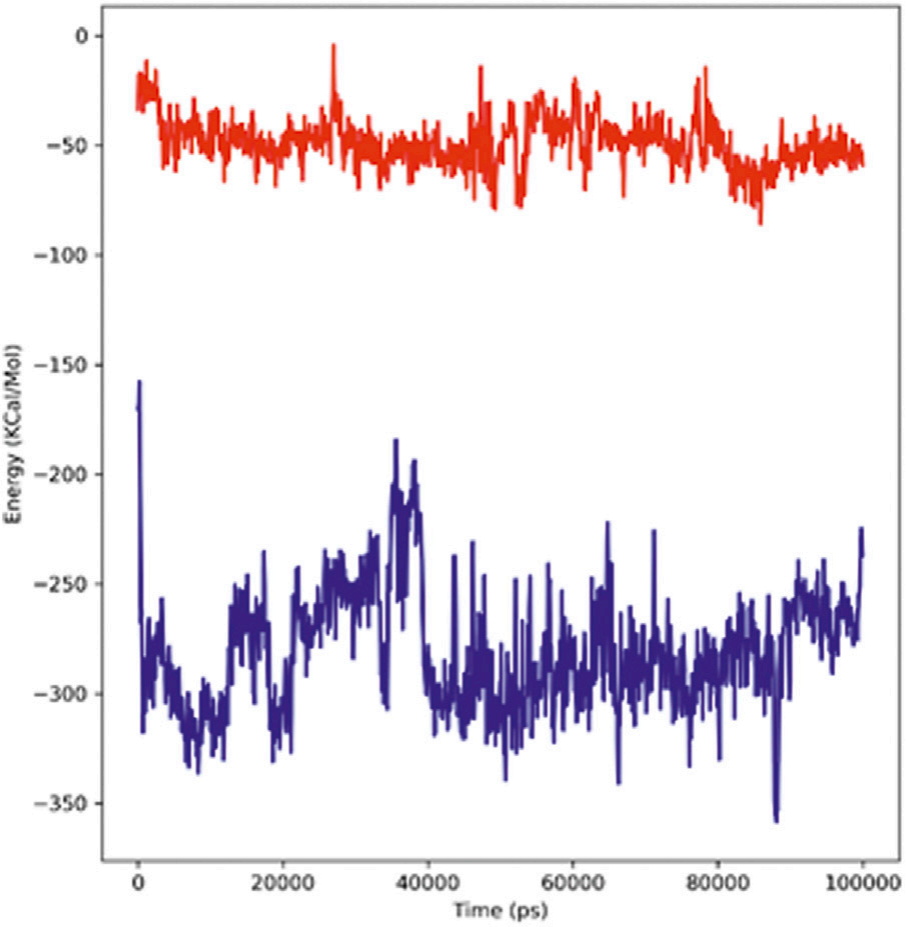
Plot representing the interactions in the form of free energies of binding between two complexes, adapalene (red) and tamsulosin (blue).

The strength with which the ligand and protein bind to each other is determined through the interaction energy calculation. The validation of binding energies generated by molecular docking analysis was performed by a detailed analysis of calculating the free energies of the interaction between the ligands and the structures of proteins. The hTERT–adapalene complex in our 100-ns simulation period showed an acceptable range of –157 to –358 kJ/mol interaction energy. The highest energy attained was –358 kJ/mol, and the average interaction energy was found to be –280.74 kJ/mol (red). The hTERT–tamsulosin complex interaction energy was found to be –69.9 kJ/mol. The average interaction energy was found to be –48.889 kJ/mol (blue). The interaction energy calculations revealed favorable binding of ligands with hTERT, validating the molecular docking results and showing the potential of ligands as drug candidates for hTERT. Figure 7 illustrates the interaction energy attained over a period of 100 ns.

#### Hydrogen bond analysis

**FIGURE 8:**
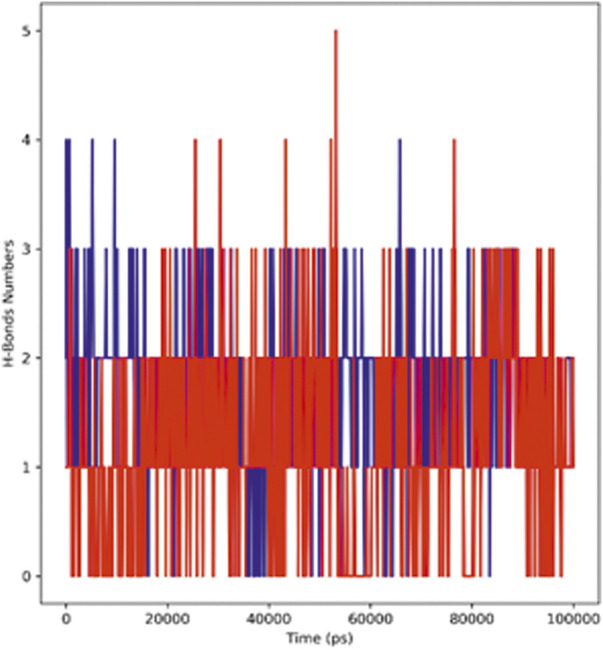
Representation of the hydrogen bonding pattern observed for protein–ligand complexes (adapalene–red and tamsulosin–blue) for 100-ns MD simulation.

Figure 8 represents the total number of hydrogen bonds formed in the complex during the simulation period of 100 ns The stability of the ligand–protein complex depends on the hydrogen bonding between the ligand and the receptor; moreover, drug specificity, metabolism, and adsorption of the drug are also dependent on hydrogen bonding. The simulation results revealed that the hTERT–adapalene complex formed four (blue) hydrogen bonds, while the hTERT–tamsulosin complex established five (red) hydrogen bonds. Overall, the details of the hydrogen bond analysis concluded effective and tight binding of ligands in the active binding site of the hTERT protein.

### *In vitro* cytotoxic assay

#### Brine shrimp assay

The brine shrimp assay is a preliminary assay to identify the cytotoxic behavior of bioactive compounds. The hatching of brine shrimps was performed at room temperature for 48 h in a simulated sea to get nauplii. Both the tested compounds, adapalene (A) and tamsulosin (T), showed considerable cytotoxic effects (Table 3).

**TABLE 3:** Brine shrimp lethality assay of tested compounds.

	Drug (A)		Drug (T)	
Concentration	% Survivors	% Death	% Survivors	% Death
Control	70	30	70	30
10 mg/ml	40	60	20	80
100 mg/ml	30	70	20	80
1000 mg/ml	0	100	0	100

#### *In vitro* cytotoxic analysis

**FIGURE 9:**
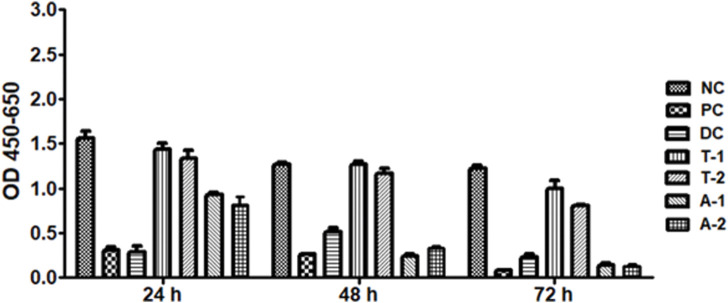
Cytotoxic analysis of the drugs adapalene (A1 and A2) and tamsulosin (T1 and T2) against HepG2 cell lines. The graph shows potential inhibition of proliferation of cells after 72 h of treatment. Graphical data are presented as mean ± SD.

**FIGURE 10:**
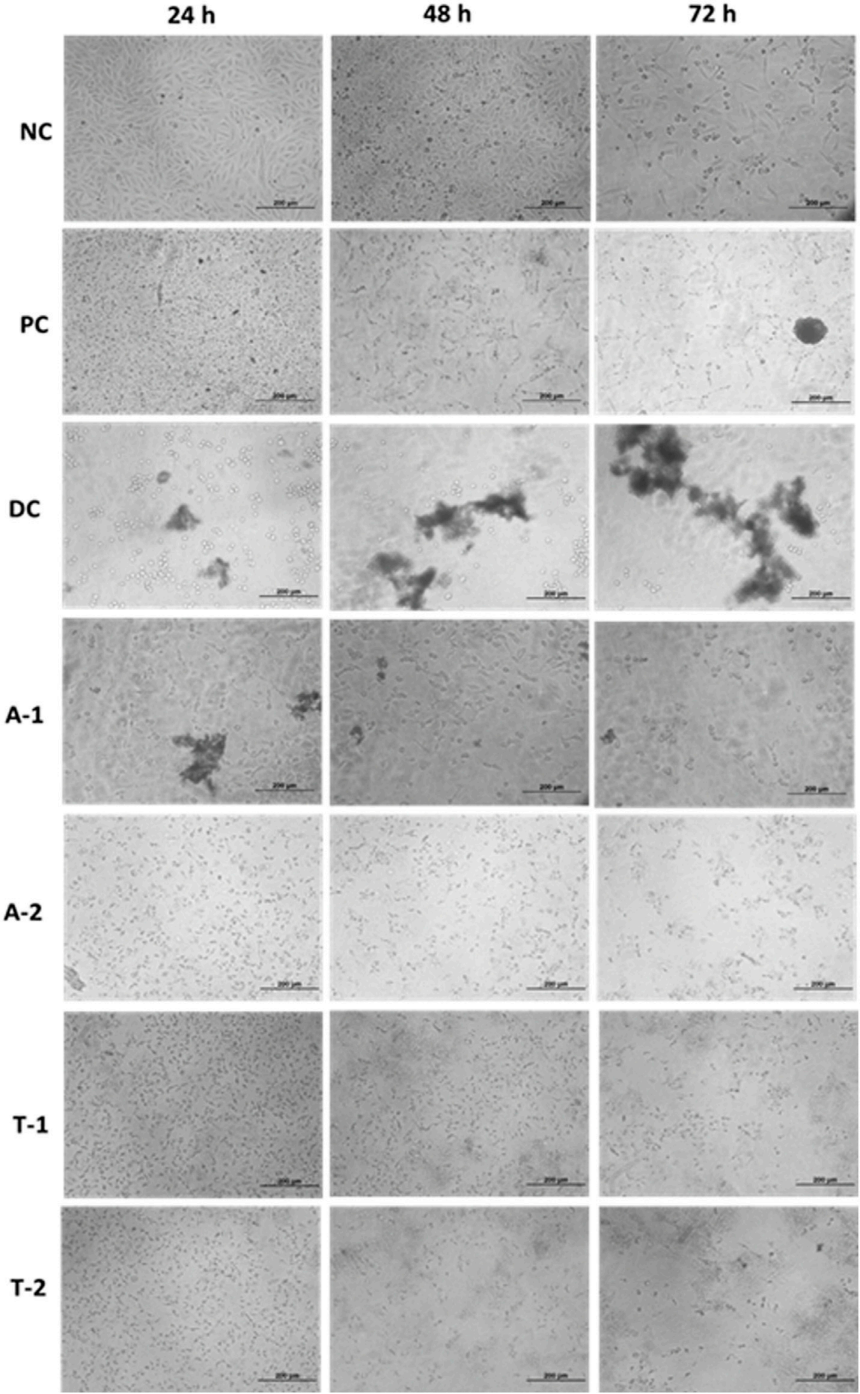
Microscopic data showing inhibition in cell proliferation over time.

Overall, the results of the XTT cell viability assay revealed the anti-cancerous activity of the given samples as the viability of HepG2 was reduced compared to NC at all-time points (24, 48, and 72 h). The drugs A1 (adapalene active) and A2 (adapalene formulation) both showed significant inhibition of cell proliferation when compared to control. The drug tamsulosin also showed inhibition at 72 h, but to a lower extent. The formulation of tamsulosin (T2) showed more inhibition when compared to the active form of tamsulosin after 72h (Figures 9, 10). The overall data showed adapalene has more potency in cellular proliferation inhibition.

## Discussion

The self-renewal of cells is basically a hallmark of carcinoma development, which is particularly regulated by the activation of telomerase. Several mechanisms have been involved in the regulation of telomerase activation, and numerous genetic and epigenetic mechanisms have shown association with its activation in different cancers. The mutations of the hTERT promoter have actually gained special attention along with the miRNA targeting of hTERT and have shown interesting properties as biomarkers. In this study, computational approaches have been utilized to screen out drugs showing potential as hTERT inhibitors. A list of FDA-approved drugs from the ZINC database was screened against the hTERT protein using a molecular docking approach. The drugs were shortlisted based on docking scores and ranked based on affinity and poses. The top two drugs with the lowest binding scores were selected for further evaluation of their hTERT inhibitory potential. Two of the FDA-approved drugs, adapalene and tamsulosin, showed the lowest binding energy of –11.1 kcal/mol. The binding analysis revealed the involvement of amino acids LEU780 for hydrogen bonding and HIS762, ARG742, PHE662, LYS659, and LEU840 for hydrophobic interactions. Similarly, tamsulosin showed interactions with PRO66, CYS54, ARG143, and TRP137. Some alkyl and pi-alkyl interactions were also observed with LEU140, LEU153, LEU152, VAL144, VAL148, VAL51, and VAL160. The molecular dynamics simulation studies were performed to analyze the binding conformation and the energy changes occurring during the ligand–binding interaction. The RMSD plot showed stability of the complexes throughout the simulations, and no sudden surge or sliding was observed. The RMSF for hTERT and two ligands were calculated, which showed the stability of the complex. The RMSF confirmed less fluctuation of protein at the binding site of ligands. However, by comparing the RMSF of the adapalene complex and tamsulosin, it was observed that tamsulosin showed less fluctuation and more stability than the hTERT–adapalene complex. The radius of gyration (Rg) for the protein–ligand complex showed similarity in both complexes, suggesting both complexes displayed similar behavior in terms of compactness. Also, the consistent value of Rg suggested good stability. The total number of hydrogen bonds involved in protein–ligand interactions was also analyzed to further understand the conformational stability. The results revealed strong hydrogen bond interactions between the protein–ligand complexes throughout the MD trajectories. The interaction energy was also calculated to evaluate the strength of protein–ligand complexes. The ligand adapalene showed the highest average interaction energy of –280.74 kJ/mol, while tamsulosin showed the highest energy of –48.89 kJ/mol. The results revealed better interaction energies for both complexes.

The selected drugs were subjected to antitumor analysis to determine whether these compounds promote inhibition of cell proliferation. In this study, two forms of drugs were used: an active form of the drug and its formulation. A mucoadhesive formulation was developed for adequate adhesiveness, spreading, and viscosity, ensuring good absorption of the drug. The formulation was developed using a combination of mucoadhesive agents, including HPMC K-15M, carbopol-934, and xanthan gum, in different ratios. The formulations were referred to as AC1, AC2, ACX1, ACX2, and ACX3 and were prepared by using Carbopol 934 and HPMCK-15M as primary polymers, while xanthan gum was used as a secondary polymer. Among all the formulations, ACX3 showed the best results based on the rheo-mechanical attributes. The adhesion time of this formulation was better than the commercially available intravaginal reference product. The preparation method and affectivity of these formulations can be found in our previous study (Afzaal et al., 2022). We planned to study the efficacy of both forms of drugs and compare whether the formulation affects the activity of the drugs. Both the formulation and the active form of adapalene and tamsulosin showed the same results. No difference in the activity between the two forms was observed, suggesting the effectiveness of the mucoadhesive formulation of these drugs.

The antitumor activity was evaluated using the cell line HepG2. Numerous studies have reported the exhibition of the telomerase activity in the majority of hepatocellular carcinomas (HCCs). The studies revealed the relation between the upregulation of hTERT expression and the level of the telomerase activity (Saini et al., 2009; Chen et al., 2013). The overexpression of hTERT and telomerase activity in HepG2 cancer cells has also been reported in another study, with the highest expression observed in G_1_/S- and S-phases (Murofushi et al., 2006). By inhibiting the telomerase activity of tumor cells, tumor cell growth, proliferation, invasion, and metastasis can be reduced. Guo and coworkers identified the anti-proliferative effect of *Atractylis lancea* (*Thunb*.) (AL) in HepG2 cells by the downregulation of the c-myc/hTERT/telomerase pathway. AL also showed the diminished telomerase activity caused by mRNA inhibition and hTERT and c-myc protein expression. Their study confirmed the role of the c-myc/hTERT/telomerase pathway in the proliferation of Hep-G2 cells (Guo et al., 2013). The anti-proliferative effect on HepG-2 cells through telomerase inhibition was also investigated by Noureini *et al.* Their study reported decreased activity of telomerase in HepG2 cells after treating them with crocin. The downregulation of the enzymatic expression of catalytic subunits was observed, which probably accounted for the anti-proliferative effect of crocin (Noureini and Wink, 2012). The pathway through which hTERT protein synthesis is regulated in HepG2 cells was also studied by Zuo and co-workers. They identified the role of NF-kappaB on hTERT synthesis in the HepG2 cells. Their findings reported the modulation of hTERT mRNA levels by NF-kappaB (Zuo et al., 2011). In one of the studies, a new siRNA was found to be effective against hTERT gene silencing in five human cancer cells, including the HepG2 cells. The study reported that the transfection of SS-PEI/hTERT siRNA induced low levels of hTERT mRNA and protein and reduced telomerase inhibition of cell growth and significant cell apoptosis. The study supported the role of hTERT protein expression in the proliferation of hepatocellular carcinoma (Xia et al., 2012). Based on the evidence of the role of hTERT protein in the proliferation of HepG2 cells, we evaluated the effects of adapalene and tamsulosin on the cellular proliferation of HepG2 cell lines. The results revealed that both compounds showed inhibition after 72 h of treatment. Adapalene showed more significant inhibition than tamsulosin when compared to the positive control. Moreover, the formulation of adapalene (A2) showed more inhibition than the active form of adapalene after 48 h; however, at 72 h the inhibition by A2 was increased (Figures 9, 10). Adapalene belongs to a third-generation synthetic retinoid and has potential use as topical therapy for acne vulgaris. The anti-tumor potential of adapalene was first explored by Ocker et al. who demonstrated its role in colorectal cancer (Ocker et al., 2003; Ocker et al., 2004; Shi et al., 2015). Other than colorectal cancer, the effect of adapalene on hepatoma cells (HegpG2 and Hep1B) was also studied by Ocker and coworkers. Their results showed inhibition of hepatoma cell growth by adapalene *in vitro,* suggesting it is an inducer of apoptosis in hepatoma cells. The results are consistent with our study justifying the role of adapalene in hepatoma cell growth inhibition. Recently, the antitumor activity of adapalene in a melanoma cell line has also been investigated, and it has shown that adapalene may be a potential drug candidate for melanoma treatment (Anaya-Ruiz and Perez-Santos, 2022). Tamsulosin (T2) is an alpha-blocker with potential use in the treatment of an enlarged prostrate. The cytotoxic efficacy of tamsulosin is still not fully explored. The effect of tamsulosin on prostate cancer was studied by Kyprianou and coworkers, who demonstrated no potential role of tamsulosin in prostate cancer (Kyprianou and Benning, 2000). Our study has, for the first time, explored the anti-tumor potential of tamsulosin against HepG2 cell lines, and the results confirmed noteworthy inhibition of cell proliferation by tamsulosin when compared to the negative control. The study highlighted the importance of computational techniques in the identification of novel efficacy in already known drugs. Moreover, the simulation studies supported the stable interaction of drugs and target hTERT, and the *in vitro* analysis confirmed the effect of these drugs as anti-tumor agents. Nevertheless, we believe further testing of these drugs is required, and their *in vitro* inhibitory potential against hTERT needs to be investigated thoroughly.

## Conclusion

The study aims at identifying inhibitory molecules for the hTERT protein. For this purpose, molecular docking and molecular dynamic simulation studies were successfully performed in order to analyze the inhibitors of the hTERT protein based on the drug repurposing strategy. The screening of 2,915 sets of compounds from the ZINC database was performed by the molecular docking method. The top two hit compounds were validated for relative stability by MD simulation runs. The simulation studies at 100 ns revealed that both compounds showed structural stability during this period of simulation. The hit compounds adapalene and tamsulosin were also further subjected to antitumor activity against the hepG2 cell line, and the results revealed promising antitumor activities of both compounds, with adapalene showing more inhibition than the control. The detailed analysis revealed the important role of *in silico* approaches in providing potential repurposing drug candidates.

## Data Availability

The original contributions presented in the study are included in the article/Supplementary Material; further inquiries can be directed to the corresponding authors.
